# The carry-over effect of an aquatic-based intervention in children with cerebral palsy

**DOI:** 10.4102/ajod.v7i0.361

**Published:** 2018-10-29

**Authors:** Samantha J. Ballington, Rowena Naidoo

**Affiliations:** 1 Discipline of Biokinetics, Exercise and Leisure Sciences, University of KwaZulu-Natal, South Africa

## Abstract

**Background:**

Cerebral palsy (CP) is the most common motor disability in childhood. Children with CP are more likely to have lower levels of physical activity than their peers, which has negative implications for their health. However, aquatic exercise can be used to improve levels of fitness among children with CP.

**Objective:**

To determine the carry-over effect of an aquatic-based programme (postural control and balance) on land (walking, running and jumping) in children with CP, post aquatic intervention.

**Method:**

The study used a pretest-post-test, randomised group, cross-over design. Children aged 8–12 years (*n* = 10) were divided into intervention (*n* = 5) and control (*n* = 5) groups. The intervention group participated in two 30-min sessions a week, while the control group continued with normal activities. Pre- and post-intervention testing was conducted using gross motor function measurement. The 10-point programme of the Halliwick Concept was used.

**Results:**

Results demonstrated that the aquatic therapy had a significant effect on gross motor function scores. The aquatic programme-based group showed increased motor function following the intervention, compared to the control group (*z* = -2.803, *p* = 0.005). Furthermore, the aquatic-based therapy improved the average score for gross motor function measurement, post-intervention.

**Conclusion:**

Together with conventional modes of therapy, aquatic-based programmes should be integrated and considered as an essential, ongoing mode of treatment for children with CP, in order to ensure long-term gross motor function improvements.

## Introduction

Cerebral palsy (CP) is a collective term for non-progressive motor conditions that cause physical disability because of affected muscle tone (Bax et al. 2005). Cerebral palsy is commonly classified based on movement disorders. The word ‘cerebral’ refers to the cerebrum, which is the affected area of the brain, and ‘palsy’ indicates a movement disorder.

Levels of mobility in the community, at school and at home can be described applying the Gross Motor Function Classification System (GMFCS), specifically in children (Galuppi et al. 1997). The GMFCS ranges from level I, representing high-functioning children who have the potential, or the ability, to walk without limitations; to Level V, representing children with very limited mobility, requiring very high levels of mobility support (Bartlett et al. 2008).

Cerebral palsy is the most common motor disability in young children. Population-based studies worldwide have described the prevalence of CP ranging from approximately 1.5 to more than 4 per 1000 live births (Cans 2000). About one in 323 children has been diagnosed with CP, according to estimates from the Centre for Disease Control and Prevention, Autism and Developmental Disabilities Monitoring Network (Arneson et al. 2009). In Africa, CP rates have been found to be even higher, with an estimated prevalence of 2–10 cases per 1000 births (Donald et al. 2014). However, there are limited studies regarding the prevalence of CP in developing countries. Two South African studies (Christianson et al. 2002; Couper 2002) indicated high prevalence rates for CP of between 1% and 8%, respectively, but there is no recent data to date (Couper 2002). The CP Association of Eastern Cape, based in Port Elizabeth, estimates that one out of every 400 babies born each year presents with CP, totalling an estimate of 2200 children diagnosed with CP yearly in South Africa (Christianson et al. 2002).

A major problem affecting the function and health of children with CP is inadequate physical activity. The development of secondary conditions related to CP, such as fatigue, osteoporosis and chronic pain, may also contribute to the lack of sufficient physical activity (Brunstrom et al. 2007). Furthermore, restrictions in posture and movement can restrict activity and are often accompanied by disturbances in sensation, sight-based perceptual problems, depth perception, communication ability and cognition (Lepore 2011; Leviton, Paneth & Rosenbaum 2007).

Children with CP, across the severity spectrum, are more likely to have diminished levels of physical activity than their peers; this increases their chance of developing other adverse health conditions such as obesity and cardiovascular disease (Leviton et al. 2007). They may only be capable of limited movement, reducing their amount of natural day-to-day exercise. Furthermore, children with CP take part less in active, physical play and playground activities. They spend more time sitting and take fewer steps per day than other children without disabilities (King, King & Law 2006). These low levels of physical activity are indicative of low endurance and a lack of general physical condition (Brunstrom et al. 2007). Moreover, because of muscle weakness, poor joint alignment and contractures, children with CP are at risk of developing overuse syndromes and other chronic conditions (Kelly & Darah 2005). Hence, early diagnosis and appropriate therapy are essential for the rehabilitation of children with CP (Dimitrijević & Jakubi 2005).

Cardiorespiratory endurance is an important element of physical fitness and has been identified as the most important fitness component linked with health and mortality (Blimkie, Malina & Strong 2005). Children with CP present with low cardiorespiratory endurance, which may be limited by their cardiovascular, respiratory and skeletal muscle systems (Brunstrom et al. 2007). With growing evidence suggesting that children with CP have reduced levels of cardiorespiratory endurance compared with children of the same age group without CP, physical therapists have recognised improving aerobic fitness as an important rehabilitation objective (Kelly & Darah 2005).

Preliminary research suggests that aerobic exercise programmes may increase cardiorespiratory endurance and other physiological responses in children and young people with CP (Berg 1970; Speth, Van Baak & Van Den Berg-Emons 1998). Although aerobic exercise has produced encouraging physiological outcomes in children with CP, the properties of activity and involvement components are unknown (Brinks, Fuler & Rogers 2008).

Exercise as a form of therapy and rehabilitation is essential for children with CP, as it is used to improve muscle strength, flexibility, respiratory function and children’s gross motor function (Keyes & Lockette 1994). Children and adolescents with CP have weak muscles; therefore, it is a reasonable assumption that these weak muscles can be strengthened via a resistance training protocol. However, a systematic review of randomised controlled trials has not shown resistance training to be significantly more effective than no intervention or placebo (Scianni et al. 2009). This is further supported by a review conducted by Verschuren et al. (2011), who also reported that there was limited evidence from land-based programmes that strength improvements correlate with improvements in activity, as the carry-over effect is generally low or absent.

Kelly and Darah (2005) believed that by being in the water, children are motivated to move their bodies and feel the effect of movement on their body. Therefore, aquatic exercise can be used to enhance the level of fitness among children with CP. The possible benefits of adaptive aquatic programmes include an improvement in cardiorespiratory endurance, strength, co-ordination and swimming skills (Fragala-Pinkham, Haley & O’Neil 2010).

Buoyancy is one of the physical properties of water which offers postural support and reduces loading on unstable joints, to allow children to move more independently in water than on land (Kelly & Darah 2005). Unrestrained movement and the ability to activate muscles that have difficulty in overcoming gravitational restrictions are the principal reasons why swimming and any aquatic-related activities are appropriate for individuals with a wide range of physically disabling conditions (Prins 2009).

Movements are completed more easily in aquatic-based exercise programmes than land-based exercise programmes. It is believed that modified aquatic-based exercise programmes transfer active exercises that are normally performed on land to the medium of water. Furthermore, the aquatic-based exercise can produce a carry-over effect on land. For example, a person who is incapable of walking on land may be able to walk in water, thereby strengthening the muscles required for walking on land (Lepore, Gayle & Stevens 2007), leading, in due course, to improved walking on land.

As CP causes a permanent disorder of movement and posture (Leviton et al. 2007), it is imperative for programmes to include a substantial number of muscle strength components to increase postural stability and prevent secondary musculoskeletal deficiencies. If muscle strength can be improved in water, it is anticipated that this may translate into improved movement on land and, in turn, better functional ability in daily living. However, the lack of aquatic-based activity programmes for this population, and the effectiveness of such interventions for children with CP, has not been well evaluated (Verschuren et al. 2011), hence the need for further investigations.

Studies including aquatic-based programmes or activities as part of therapy with ambulatory children and adolescents with CP, classified with gross motor function at levels I, II and III, are few and are even more limited on children classified with gross motor function at levels IV and V (Blohm 2011; Currie & Gorter 2011; Dimitrijevic & Jorgic 2012; Franzen & Tryniszewski 2013). Furthermore, relatively low sample sizes, ranging from 1 to 16 participants, were used, with the majority of studies (Currie & Gorter 2011; Franzen & Tryniszewski 2013) recruiting < 7 participants or being implemented as single-subject case studies. Personal and environmental barriers such as acceptance, fear, transportation, time and accessibility may play a role in low participation and study completion (Viguers 2010).

Thus, further research on the carry-over effect from the aquatic environment to activity on land is required (Verschuren et al. 2011). The aim of this study was to determine the carry-over effect of an aquatic-based programme (postural control and balance) to movement on land (walking, running and jumping) in children with CP, using pre- and post-intervention measurements.

## Research methods

### Research design

The study used a pretest-post-test, randomised group, cross-over design. Taking into consideration the population in this study, it is difficult to recruit a large sample. A cross-over design requires fewer participants in order to attain the same level of statistical significance or precision as a parallel design. Therefore, the cross-over design was warranted as this design yields a more efficient comparison of treatments than a parallel design (Chen et al. 2009).

### Participants

Participants were recruited on a voluntary basis from a local school in the eThekwini area, KwaZulu-Natal, South Africa. To participate in the study, children had to fulfil the following inclusion criteria: they must have been medically diagnosed with CP; have a GMFCS score of between I and III; and would have no other medical conditions, such as seizures. The exclusion criteria were the following: a GMFCS score of more than III and having other medical conditions that caution against water therapy. Based on the inclusion and exclusion criteria, a sample of 10 children (2 males and 8 females) between the ages of 8 and 12 years, with a mean age of 11 ± 0.08 years, was selected to participate in the study.

### Testing procedures and protocol

Parental consent and child assent were obtained on an individual basis. The study was approved by the Biomedical Research Ethics Committee of the University of KwaZulu-Natal. Participation was voluntary and participants could withdraw from the study at any time.

Children were randomly divided into an intervention (*n* = 5) and a control (*n* = 5) group. Pre- and post-intervention gross motor function testing was conducted individually at the school during school hours on all the children. The gross motor function measurement (GMFM) testing protocol was used.

The intervention group participated in two aquatic sessions a week for 8 weeks, so a total of 16 sessions. Each session lasted 30 min. The control group did not participate in any aquatic-based activities during this part of the study. At the end of the 8 weeks, post-intervention tests were conducted prior to the wash-out period of 1 month (school vacation). Thereafter, there was a cross-over between the groups, with the control group now participating in the same aquatic-based activities that the intervention group had performed for a period of 8 weeks, while the previous intervention group now continued with normal activities.

### The aquatic-based intervention

The 10-point programme of the Halliwick Concept was used. This included water adjustment skills, longitudinal rotations, sagittal rotations and swimming skills. Each session consisted of a one-on-one session with a qualified biokineticist (a specialised exercise therapist who functions in professional alliance with health and medicine and is recognised by, and registered with, the Health Professions Council of South Africa (HPCSA 2016)). The 30-min session comprised a 5-min warm up, followed by a 20-min session based on the Halliwick Concept and ended with a 5-min cool down. The warm-up session for the first session was water orientation, allowing the children to get used to the water, and thereafter, as a warm up, a quick recap of the previous session was conducted, before moving onto the next point in the programme. The cool-down session consisted of free play. This included splashing and jumping in the water as well as diving down under the water.

The Halliwick Concept is a detailed swimming programme-based on the scientific principles of body mechanics and the properties of water intended to educate individuals with special needs to be water safe and to move independently in the water as much as possible (Lambeck & Stanat 2001a). The programme consists of 10 specific progressive stages that are achieved without the use of floatation devices (Lambeck & Stanat 2001b). These 10 points have been ordered to provide a universal structure, although there is a strong overlap between the points (IHA 2010). Furthermore, through the 10 points, a process of development through balance control, mental adjustment and movement leads to individual independence in the water. These three concepts are the necessary mechanisms of motor learning (Lambeck & Stanat 2001a; 2001b).

### The 10-point programme of the Halliwick Concept

The 10-point programme consists of the following points (IHA 2010):

- **Point 1**: *Mental adjustment* is the process which allows the swimmer to be in the water with sufficient confidence to experience water in a positive way. It includes learning to blow out or hum when the mouth or nose comes in contact with the water.
- **Point 2**: *Disengagement* is the process through which swimmers further develop their confidence and which allows them to start exploring the environment, moving away from the poolside, pool floor or the support of the therapist.
- **Point 3**: *Transversal rotation control* is controlling rotation around a transversal axis. For example, the sequence of floating on the back to reaching a vertical position in the water, pivoting around an axis which passes through both hips.
- **Point 4**: *Sagittal rotation control* is controlling rotations around a sagittal axis. For example, remaining vertical when reaching for an object placed to the side of the body and preventing pivoting around an axis perpendicular to the frontal plane of the body.
- **Point 5**: *Longitudinal rotation control* is controlling rotational movements taking place around a longitudinal axis. For example, preventing the rotation to the right side generated when turning the head to the right while floating horizontally on the back. In this example, the person is preventing rotation around an axis perpendicular to a transversal plane.
- **Point 6**: Combined rotation control is controlling any combinations of the above described rotations. At this point, the swimmer initiates or prevents several rotations at once. For example, moving forward from a vertical position to achieve a position floating on the back.
- **Point 7**: *Up-thrust* is when the swimmer learns that the water can help him or her to stay at the surface. Having this experience increases the swimmer’s confidence to cope with less, or no, support.
- **Point 8**: *Balance in stillness* is about developing the ability to respond in a controlled way when unsupported in the water and balance is challenged.
- **Point 9**: *Turbulent gliding* is the swimmer moving through water with no direct support from the instructor and without making propulsive movements. For example, in a back float, the swimmer’s body is in motion thanks to the turbulence generated by the instructor’s hands and/or body. This helps the swimmer to maintain balance in stillness while experiencing increasing forces disturbing the position of his body in the water.
- **Point 10**: *Simple progression or basic swimming strokes* is about using simple movements to create propulsion; for example, clapping the hands on the thighs when in a back float to propel the body through the water. From the use of simple movements, more sophisticated swimming movements or strokes can be learned.

### Gross motor function measurement-66

The GMFM-66 is a standardised observational instrument designed to measure changes in gross motor function over time in children with CP. The GMFM assesses motor function (how much the child can do) opposed to the quality of the motor performance (how well the child can do) (Palisano & Russell 1998).

The 66 items on the GMFM-66 span the spectrum of activities, from lying and rolling-up, to walking, running and jumping skills. The GMFM provides detailed information on the level of difficulty of each item. It is a comprehensive evaluation of foundational gross motor skills, is responsive to change which makes it an ideal pre- or post-measurement and is useful for setting goals and planning interventions in therapy (Bartlett et al. 2008). Each child performed the test items without shoes, wearing shorts and a T-shirt, to ensure standardisation during testing.

### Data analysis

The GMFM-66 employs a four-point scoring system for each item. The 66 items are recorded according to the following classification: 0 = child unable to initiate the task; 1 = child initiates the task; 2 = child partially completes the task; 3 = child completes the task; and *NT* = not tested. Each individual score was thereafter entered into a computer programme called the Gross Motor Ability Estimator. Individual item scores for all 66 tests were then converted to an interval-level total score. An interval-level measurement of gross motor function based on a child’s score on the items of the GMFM was calculated. The computer scoring can be used to track scores over time and also generates item maps. These maps can inform the user which skills the child is likely to achieve next, which may be helpful for therapy.

Statistical analysis was performed using the Statistical Package for the Social Sciences (SPSS) software (version 21.0 for Windows; IBM, Armonk, NY, USA). Alpha level was set at *p* < 0.05. The scores from the GMFM were compared pre- and post-intervention using the Mann-Whitney test. The paired *t*-test and the Wilcoxon signed rank test were also used to determine significant differences pre- and post-intervention. A limitation of the GMFM score is that it gives no normative data for the total GMFM score. However, the GMFM is used as an observational tool, comparing pre- and post-test score improvements (Avery et al. 2000).

### Ethical considerations

Parental consent and child assent were obtained on an individual basis. The study was approved by the Biomedical Research Ethics Committee of the university (BF201/15). Participation was voluntary and the participants were informed that they could withdraw from the study at any time.

## Results

Pre-intervention and post-intervention included a 100% compliance from the 10 children for the duration of the study.

During the intervention, each child began the Halliwick Concept at Point 1 of the 10-point programme, gradually progressing to the next point at their own comfortable speed. However, two children encountered difficulty with the first two points. This was primarily because of this being their first time in a swimming pool. Thus, the two children did not complete the 10-point programme within the 8-week intervention period. The remaining eight children completed the 10-point programme within the allocated 8-week intervention period.

Pre- and post-test results demonstrated that the aquatic-based intervention had a significant effect on the GMFM-66 item scores. The intervention group’s post-intervention score increased more (*z* = -2.803, *p* = 0.005) than the control group’s score. Furthermore, the intervention group’s average score on the 66-item GMFM increased by 4.25 points.

When applied to each group separately, to test whether the intervention had an effect, both groups showed gains following the aquatic-based intervention which were significantly greater than following no activity (*z* = -2.805, *p* = 0.005).

As the study had a cross-over design, groups can be further divided into intervention and control and control and intervention. There was a significant difference in both intervention and control and control and intervention groups pre- and post-aquatic-based intervention (*z* = -2.023, *p* = 0.043 and *z* = -2.023, *p* = 0.043, respectively). Additionally, to calculate the average of the post-intervention group and the post-control group scores, a Mann-Whitney test was applied. Results showed that the wash-out period was long enough for the effect from the intervention not to be carried over when the groups crossed over.

During the study, the researcher noted the following incidental findings. During pretests using the GMFM, the children performed several tasks, for example, walking up and down four steps with and without holding the rail. Selected children, based on their past experiences, were unable to accomplish a selected task or tasks and therefore verbally declined to attempt the task. Similarly, during the post-intervention testing, the same children once again verbally declined to attempt the same task, because of their perceived knowledge and attitude that they could not perform the task. Moreover, the children were afraid to attempt the task, primarily because of fear of failure and/or injury.

However, one of the children, during the pre-intervention testing, declined to walk up and down the steps, because of past experiences and an inability to adequately lift his or her feet off the ground. During the intervention, the child was able to walk correctly and lift his or her feet in the water, a movement that had not been possible previously. This, in turn, encouraged the child to perform all the assessments of the GMFM post-intervention testing.

## Discussion

This study investigated the carry-over effect of an aquatic-based intervention to land in children with CP. The aquatic-based intervention used the Halliwick Concept. Unlike most aquatic programmes (Fragala-Pinkham, Haley & O’Neil 2008; Fragala-Pinkham et al. 2010) that mimic land-based activities, the Halliwick incorporates rotational movements, balance and floating, hence facilitating sensory input (Lambeck & Gamper 2010). Additionally, the static component promotes the activation of selective muscles and the stabilisation of specific joints (Lambeck & Gamper 2010). Many of the activities can be repeated and varied, which teaches balance strategies that can provide carry-over effects to land (Getz, Hutzler & Vermeer 2006).

The Halliwick Concept has been used in studies on children with CP to examine its effect of neurological conditions. Hou, Wan and Li (2010) found that the conventional rehabilitation, together with the Halliwick Concept, had significant effectiveness on the gait functions of children with spastic CP. Similarly, this study uses the Halliwick Concept as part of an aquatic-based intervention to produce a carry-over effect, that is, improved gross motor function reflected in improved walking, running and jumping on land.

These findings suggest that child aquatic therapy can improve motor function in children with CP, even in children classified with gross motor function at level III, who are restricted in their ability to perform land-based activities. The aquatic-based intervention showed beneficial effects on the gross motor function of the children, as the average 66-item GMFM score increased by 4.25 points. According to Wang and Yang (2006), 66-item GMFM scores of > 3.7 show great improvement; scores of 1.6 – 3.6 indicate clinically significant improvement and scores < 1.6 indicate no clinically significant improvement.

However, one should be cautious about claiming significant improvements, particularly after a short period of time. In this study, statistical analysis showed that the 1-month wash-out period was long enough for the effect from the intervention not to be carried over when the groups crossed over. Therefore, the positive effects of the intervention were only short-term.

Furthermore, a possible reason for the increase in the 66-item GMFM score may be the thermal and mechanical effects of aquatic-based exercises (Lai, Lui & Yang 2015). The thermal properties are helpful for pain and spasticity decreases. The mechanical properties offer benefits by decreasing the effect of gravity and joint loading, and assisting with postural support and muscular strength. The water viscosity extends falling time and allows the participants to experience movement patterns that allow their centre of gravity to be temporarily outside the base of support without the fear of falling. These factors have been credited with an increase in performance, such as neuromuscular co-ordination, muscular endurance and aerobic capacity (Fragala-Pinkham et al. 2008). Additionally, the relaxing effect and the increased body weight support of the aquatic environment may have facilitated a reduction of spasticity and an increase in muscular strength, thus allowing the child to initiate movements that are restricted on land. This, in turn, allows for an improvement in postural control, balance and walking on land (Becker 2009; Currie & Gorter 2011).

An aquatic environment can provide benefits not achievable on land for children with CP who require reduced compressive loads on joints in order to achieve voluntary movement, and it can also be a fun environment filled with opportunity for improved sensory stimulation (Barlow et al. 2009). In the water, the limiting movements of the condition appear less apparent in skill functions (Heckathorn 1980). Water is known to be an acceptable medium for treatment to relax abnormal muscle tone (Harris 1978). The Halliwick Concept progressively teaches independence in water, a prerequisite for participation in therapeutic, recreational and vocational activities individually or in a group (Lepore et al. 2007). On reaching point 10 of the Halliwick Concept, the child has the ability to perform basic swimming strokes. Swimming has many psychodynamic aspects. It requires active movement, it is a structured activity and the participant can progress and note personal achievements (Dumas 2001). The authors believe that with the combined expertise of the therapist, an activity such as swimming may be used to help achieve therapeutic goals, as well as recreational goals within a therapy programme, for children with CP. Swimming may be perceived by the child as a recreational or sporting activity, not as therapy, and this may promote a sense of ‘normality’.

However, if the Halliwick Concept is utilised as a treatment technique, it is imperative not to progress too rapidly through each point. The authors believe that Point 1 (mental adjustment) in the Halliwick Concept is the most vital component and is the basis for the programme progression. A therapist should ensure that the child has gained sufficient confidence in water and that any fear of the water is overcome, before introducing new concepts. In this study, 2 children had not previously been exposed to aquatic therapy because of a perceived fear of the water. Therefore, additional time was spent on Point 1 of the Halliwick Concept, by gradually attempting to change their perceived fear of the water, that is, the mental adjustment. Although, these 2 children did not complete the 10-point programme at the end of the 8 weeks, the authors believe that if the intervention period had been extended, the children would have completed points 9 and 10. The remaining 8 children progressed through the intervention programme as per Halliwick Concept design.

The ability to perform movements more easily in water promotes a level of control and independence, which many people with disabilities cannot achieve on land (Lepore et al. 2007). This study found that this control and independence achieved by the children was converted into a feeling that fuelled self-confidence. This self-confidence in turn appeared to alter the children’s perceptions and attitudes towards their personal capabilities. Movements that children were unable, and/or perceived themselves unable, to perform on land were performed in the water. Subsequently, with an increase in self-confidence, selected movements and movement patterns were carried over to land from the water. Geralis (ed. 1998) believed that achievements in the water can lead to increased confidence. Self-awareness and self-esteem will also improve as the child develops the ability to move and enjoy the water.

Aquatic-based exercises have also been found to promote pleasure, providing a chance to increase the enthusiasm of the children and aiding the motor development in children (Lai et al. 2015). Hence, an aquatic-based exercise programme, like in this study, can be conducted as an effective substitute for, or complementary treatment to, the conservative therapy for children with CP.

A therapist should always aim to adopt reliable and valid activities or techniques to incorporate within treatment modalities. However, it is important to assess the patient’s response to that particular treatment in order to develop a successful programme. The individual’s co-operation and involvement is essential in treatment planning (Currie & Gorter 2011). Children have been found to respond positively to activities presented in a non-threatening manner. The direct participation of staff or family members in activities may help encourage the child and meet the developmental needs of the child (Kellaghan, Sloane, Alvarez & Bloom 1993). Particularly with the inclusion of an aquatic component, support from parents, teachers and other health professionals is vital. In this study, part of its success can be attributed to the support from parents and school teachers, as well as the physiotherapist and occupational therapist who worked regularly with the children. Although, in this case, direct participation was not permitted during the intervention sessions, their positive attitude to participation and encouragement of the children in the programme was noted.

The level of enjoyment of the programme used in this study was not measured. However, it was clear that the aquatic based-programme seemed to be enjoyable for the children, which ensured their participation and the completion of the intervention.

Overall, this study has shown that an aquatic-based intervention is physically beneficial to children with CP. It was observed that the improved effectiveness of the aquatic-based programme was primarily based on the ability of the child to participate at a high comfort level in the water, thus enabling the completion of all activities. The ability to move freely in the aquatic environment at the beginning of the programme seemed to be beneficial to the children. The children were motivated to participate in activities and exercises for the 16 intervention sessions.

Lastly, findings from this study provide a basis for further research in areas of aquatic-based activity and physical activity in children with CP. There is still a need for well-designed intervention studies with adequate sample sizes in a population with a wider range of severity levels, although GMFCS levels IV and V can be challenging. Forthcoming studies should also vary aquatic therapy protocols, such as community-based group therapy, family participation and longer intervention periods. Another recommendation would be to measure the effects of aquatic-based therapy, progressing to a land-based therapy versus land-based therapy only. Studies should include muscle activation through electromyography (EMG) techniques. Such imaging can be used pre- and post-intervention, as well as pre and post each aquatic session. An important aim of the Halliwick Concept is muscle activation for postural gains and balance in the water, which is carried over to land. Therefore, by measuring the carry-over effect of aquatic-based programmes to land-based programmes, it can help with assessing the effectiveness of treatment plans for children with CP. Additionally, a questionnaire to record the child’s enjoyment of, interest in, motivation for and confidence exercising in the water may offer information on additional factors which could influence future rehabilitation programmes.

## Limitations of the study

This study had several limitations. Firstly, it had a small number of participants, and therefore, the possibility of extrapolating to other populations is limited. Secondly, the participants did not have a broad range of severity levels: no participants had gross motor function classified at GMFCS levels IV and V. Thirdly, the study was designed as aquatic-based exercise only and determined the carry-over from water to land, but did not determine the effect of land-based exercise only. Fourthly, the study was conducted over 8 weekly sessions for each participant, which put a time restraint on some participants as they did not finish the whole 10-point programme. And lastly, a questionnaire to assess the enjoyment and or psychological status of the participants, which would have strengthened the overall study design, is warranted in future studies.

## Conclusion

In conclusion, an 8-week aquatic-based intervention has the potential to produce greater gains in gross motor function in children with CP, also producing a significant carry-over effect onto land. However, this study has shown that after a month of no aquatic activity, gains in gross motor function are reversible. Therefore, it suggested that aquatic-based programmes should be integrated and considered as an essential continuous mode of treatment for children with CP, to ensure long-term improvements in gross motor function. Moreover, aquatic therapy is an innovative therapy for children with marked motor impairments, as movements in land-based exercises are restricted for this population.
